# A314 UNDIFFERENTIATED PANCREATIC CARCINOMA WITH OSTEOCLAST-LIKE GIANT CELLS: A CASE REPORT

**DOI:** 10.1093/jcag/gwad061.314

**Published:** 2024-02-14

**Authors:** M K Parvizian, D Hurlbut, M S Rai

**Affiliations:** Queen's University, Kingston, ON, Canada; Queen's University, Kingston, ON, Canada; Queen's University, Kingston, ON, Canada

## Abstract

**Background:**

Pancreatic undifferentiated carcinoma with osteoclast-like giant cells (UCOGC) is a rare form of pancreatic cancer representing under 1% of cases. UCOGC is hypothesized to be a variant of pancreatic adenocarcinoma and while the pathophysiology is incompletely defined, it is felt to be an undifferentiated sarcomatoid carcinoma variant with chemotaxis of osteoclastic giant cells. Clinical, radiographic, or biochemical features do not reliably differentiate this from other tumors. Definitive diagnosis is made by histologic examination demonstrating non-neoplastic multinucleated osteoclastic giant cells (CD68 positive), mononuclear histiocytes, and neoplastic mononuclear cells.

**Aims:**

To describe the case of a patient with UCOGC, highlighting key clinicopathologic features and management.

**Methods:**

Case report and literature review.

**Results:**

A 79-year-old man with atrial fibrillation on Xarelto presented with 4 months of abdominal pain, nausea, vomiting, and weight loss (22 kg). An MRI revealed a dilated main pancreatic duct and side branches with abrupt cutoff in the neck due to an ill-defined lesion. Endoscopic ultrasound confirmed a 36 mm hypoechoic ill-defined mass in the pancreatic neck. Biopsy performed with a 22-gauge needle with dry suction showed tissue fragments consistent with UCOGC (**Figure 1**).

Unfortunately, the patient presented to hospital with worsened abdominal pain and nausea and was found to have new portal, superior mesenteric, and splenic vein thrombosis. His anticoagulant was changed to Dalteparin. A multidisciplinary cancer conference was held including input from experts in Toronto, and due to surrounding inflammation and new thrombosis he was not considered a surgical candidate at the time and neoadjuvant treatment with standard adenocarcinoma chemotherapy was planned.

Unfortunately, the patient continued to decline and he was treated with palliative intent FOLFIRINOX for 2 cycles before opting for a medical assistance in dying procedure.

**Conclusions:**

Treatment for UCOGC has not been well studied due to its rarity. Surgery is first-line therapy if appropriate based on patient status and cancer stage. The efficacy of chemotherapy and radiation is unclear but as a suspected adenocarcinoma variant, standard chemotherapy regimens are often used. Research is ongoing with an area of interest being PD-L1 inhibition due to high tumor PD-L1 positivity rates. Recognition of this rare entity is crucial due to its distinct diagnostic and therapeutic characteristics with more research needed to establish optimal therapy.

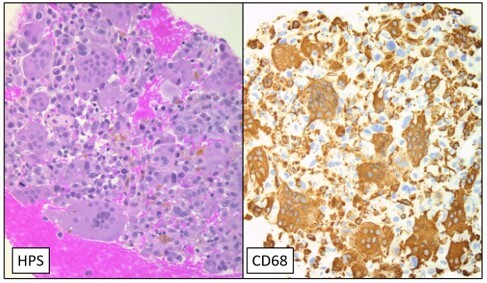

**Figure 1:** Pathologic examination with HPS and CD68 stains demonstrating UCOGC

**Funding Agencies:**

None

